# Lead‐free Double Perovskite Cs_2_AgIn_0.9_Bi_0.1_Cl_6_ Quantum Dots for White Light‐Emitting Diodes

**DOI:** 10.1002/advs.202102895

**Published:** 2021-11-28

**Authors:** Yuqing Zhang, Zehao Zhang, Wenjin Yu, Yong He, Zhijian Chen, Lixin Xiao, Jun‐jie Shi, Xuan Guo, Shufeng Wang, Bo Qu

**Affiliations:** ^1^ State Key Laboratory for Artificial Microstructures and Mesoscopic Physics Department of Physics Peking University Beijing 100871 P. R. China; ^2^ Key Laboratory of Optoelectronic Science and Technology for Medicine of Ministry of Education Fujian Provincial Key Laboratory for Photonics Technology Fujian Normal University Fuzhou 350007 P. R. China

**Keywords:** double perovskites, electrically excited, lead‐free, quantum dots, white light‐emitting diodes

## Abstract

Perovskite‐based optoelectronic devices have attracted considerable attention owing to their excellent device performances and facile solution processing. However, the toxicity and intrinsic instability of lead‐based perovskites have limited their commercial development. Moreover, the provision of an efficient white emission from a single perovskite layer is challenging. Here, novel electrically excited white light‐emitting diodes (WLEDs) based on lead‐free double perovskite Cs_2_AgIn_0.9_Bi_0.1_Cl_6_ quantum dots (QDs) without any phosphor are fabricated for the first time. Density functional theory calculations are carried out to clarify the mechanism of absorption and recombination in Cs_2_AgIn_0.9_Bi_0.1_Cl_6_ with Bi‐doping breaking the parity‐forbidden transition of the direct bandgap. Microzone optical and electronic characterizations reveal that the broadband emission of Cs_2_AgIn_0.9_Bi_0.1_Cl_6_ QDs originates from self‐trapped excitons, and luminescent properties are unchanged after the film deposition. The QD‐WLED exhibits excellent Commission Internationale de L'Eclairage color coordinates, correlated color temperature and relatively high color rendering index of (0.32, 0.32), 6432 K, and 94.5, respectively. The maximum luminance of 158 cd m^−2^ is achieved by triphenylphosphine oxide passivation, and this lead‐free QD‐WLED exhibits a superior stability in ambient air with a long *T*_50_ ≈48.53 min. Therefore, lead‐free perovskite Cs_2_AgIn_0.9_Bi_0.1_Cl_6_ QDs are promising candidates for use in WLEDs in the future.

## Graphical abstract

Neatly structured lead‐free double perovskite Cs_2_AgIn_0.9_Bi_0.1_Cl_6_ QDs with a photoluminescence quantum yield (PLQY) of 31.4% are synthesized through hot‐injection method. The electrically excited white light‐emitting diodes (WLEDs) based on Cs_2_AgIn_0.9_Bi_0.1_Cl_6_ QDs are fabricated for the first time, exhibiting excellent Commission Internationale de L'Eclairage (CIE) color coordinates, correlated color temperature, and a relatively high color rendering index of (0.32, 0.32), 6432 K, and 94.5, respectively.

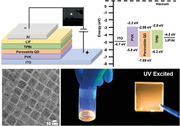

## 1 Introduction

White light‐emitting diodes (WLEDs) have been extensively investigated for applications in the display and lighting industries.^[^ ^1^ ^]^ Electrically excited devices based on red‐green‐blue units and optically excited devices formed by blue emitters with down‐shifting phosphors are the two components of most WLEDs.^[^ ^2^ ^]^ Halide perovskites exhibited considerable potentials for display, lighting and energy conversion applications, owing to their unique optoelectronic properties.^[^ ^3^ ^]^ Since the first report on WLEDs using perovskites as converting phosphors in 2015, a significant progress has been achieved in this field.^[^ ^4^ ^]^ Broadband emission of self‐trapped excitons (STEs) from single emitters with soft lattices and local carriers, such as cesium copper halides, lead‐halide perovskites, and lead‐free double perovskites, have been investigated.^[^ ^5^ ^]^ These materials can be manufactured as single crystal, bulk, two‐dimensional, and quantum dot (QD) states.^[^ ^6^ ^]^ However, no extensive studies on their electroluminescence (EL), particularly for lead‐free perovskites, have been carried out. Tang and coworkers pioneered warm‐white devices based on bulk lead‐free double perovskite Cs_2_(Ag_0.6_Na_0.4_)InCl_6_, with a maximum luminance of 50 cd m^−2^.^[^ ^5c^ ^]^ Shan and coworkers demonstrated that copper‐based ternary halide composites CsCu_2_I_3_@Cs_3_Cu_2_I_5_ exhibited cold/warm white‐light tuning features, and the corresponding WLEDs exhibited a maximum luminance of 145 cd m^−2^ and external quantum efficiency (EQE) of 0.15%, with a high color‐rendering index (CRI) of 91.6.^[^ ^7^ ^]^

Recently, researchers proposed that lead‐free double perovskite QDs with a broadband emission from STEs exhibited a high photoluminescence quantum yield (PLQY). Manna and coworkers increased the PLQY of Cs_2_AgInCl_6_ nanocrystals from 1.6% to 16% using a Mn‐doping strategy.^[^ ^8^ ^]^ Xia and coworkers boosted the PLQY of Cs_2_AgInCl_6_:Bi nanocrystals to 11.4% with trace Bi doping.^[^ ^9^ ^]^ Yella and coworkers investigated the relationship between Bi alloying and the change in bandgap structure, confirming that the bandgap of lead‐free double perovskite QDs could be tuned from indirect to direct through composition adjustment.^[^ ^10^ ^]^ Han and coworkers further verified that the series doping could influence the PLQY of lead‐free double perovskite QDs to a large extent. The reported Cs_2_AgIn_x_Bi_1‐x_Cl_6_ QDs with direct bandgap showed a high PLQY over 30%.^[^ ^11^ ^]^ Although lead‐free double perovskite QDs are suitable for WLED applications, studies on EL devices are lacking.

In this study, lead‐free double perovskite Cs_2_AgIn_0.9_Bi_0.1_Cl_6_ QDs were introduced in electrically excited QD‐based WLEDs (QD‐WLEDs) as an emissive layer for the first time. The lead‐free double perovskite Cs_2_AgIn_0.9_Bi_0.1_Cl_6_ QDs have the advantages of strong quantum confinement effect of the reduced dimension structure, emission of STEs in lead‐free materials, and superior stability of the double perovskite structure. QD‐WLEDs with competitive efficacy and brightness (EQE of 0.08%, luminance of 158 cd m^−2^) in the field of electrically excited lead‐free perovskite‐based devices were realized. Inspiringly, the obtained QD‐WLEDs were endowed with long‐term operation lifetime (*T*_50_ of 48.5 min), high CRI of 94.5, and typical white light chromaticity coordinates of (0.32, 0.32) in Commission Internationale de L'Eclairage (CIE) 1931 color space diagram. Our study will provide new insights for the application of lead‐free double perovskite QDs in lighting and display technologies.

## 2 Results and Discussion

**Figure 1:**
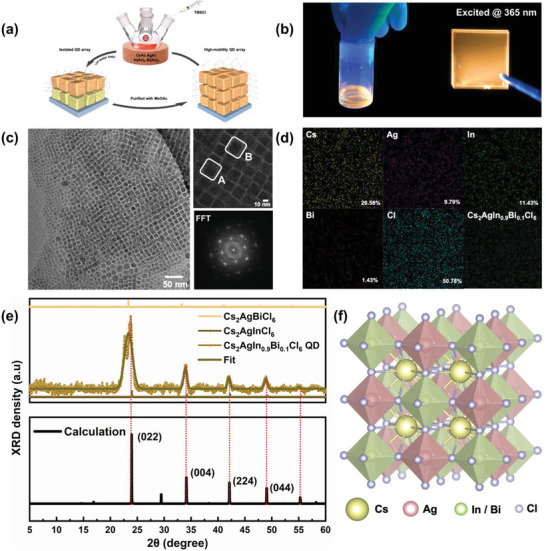
Diagram of the morphology and crystal structure characterization for Cs_2_AgIn_0.9_Bi_0.1_Cl_6_ QDs. a) Schematic of the fabrication and purification for neatly structured Cs_2_AgIn_0.9_Bi_0.1_Cl_6_ QDs. b) Photographs showing the PL properties of colloidal QDs and QD film on a fused silica substrate at an excitation wavelength of 365 nm. c) Low‐magnification and high‐magnification (inset) TEM images of the colloidal QDs, with the FFT patterns. The structure analysis of areas A and B area is presented in Figure S1 in the Supporting Information. d) Corresponding semi‐quantitative EDS composite map of the high‐magnification TEM image. e) XRD data for the QD powders (top) and theoretical simulation (bottom), which reflect the successful doping of Bi and In elements. f) Schematic crystal structure of Cs_2_AgIn_0.9_Bi_0.1_Cl_6_.

High‐quality Cs_2_AgIn_0.9_Bi_0.1_Cl_6_ QDs were prepared using a hot‐injection approach, as shown in **Figure** **1**a. Metal precursors (Cs‐, Ag‐, In‐, and Bi‐acetates) were mixed together with ligands and heated to 110 °C. The nucleation and growth of QDs were then triggered by a swift injection of the halide source. After several purification cycles via methyl acetate, neatly structured Cs_2_AgIn_0.9_Bi_0.1_Cl_6_ QDs with a bright orange emission under an ultraviolet excitation were obtained (Figure 1b; the process is presented in Supplementary Video S1). Transmission electron microscopy (TEM) images revealed that all QDs were regularly arranged uniform cubes with an average size of 9.7 ± 1.5 nm (Figure 1c). The size statistical distribution estimated from TEM images is shown in Figure S1 in the Supporting Information. It is clear that the obtained Cs_2_AgIn_0.9_Bi_0.1_Cl_6_ QDs are highly crystalline in nature. The lattice fringes correspond to (0 2 2) and (0 0 4) with spacings of 0.37 nm (area A) and 0.26 nm (area B), respectively (Figure S2, Supporting Information). The fast Fourier transform (FFT) of the TEM image reveals the cubic features of Cs_2_AgIn_0.9_Bi_0.1_Cl_6_ QDs. The energy dispersive spectroscopy (EDS) reveals the chemical composition and distribution in the QDs, where all elements are distributed evenly in the selected area (Figure 1d). A semi‐quantitative result for the ratio of In^3+^ and Bi^3+^ of 8:1 was obtained, which is close to the stoichiometric value. X‐ray photoelectron spectroscopy (XPS) was performed to verify the chemical environment of the surface elements in the QDs (Figure S3, Supporting Information). The strong Cl 2*p* signal indicates that the QDs are surrounded by halogen. A doublet is observed, corresponding to In 3*d*_5/2_ (444.8 eV) and In 3*d*_3/2_ (452.4 eV), owing to the spin−orbit splitting. Similarly, Bi 4*f*_7/2_ (156.7 eV) and Bi 4*f*_5/2_ (164.5 eV) confirm presence of trivalent Bi (3^+^). There is no shoulder peak in the In 3*d* and Bi 4*f* spectra, which implies the absence of either other valence states or metallic phases.^[^ ^10^ ^]^ The In^3+^/Bi^3+^ ratio was calculated to be 11.83 based on the integral areas of In 3*d* and Bi 4*f* peaks, which suggests that the substitution of Bi occurs at the surface.^[^ ^12^ ^]^ All Cs_2_AgIn_0.9_Bi_0.1_Cl_6_ QDs retained their double perovskite structure with a space group of $Fm\bar{3}m$, as shown in the X‐ray diffraction (XRD) patterns (Figure 1e). A schematic of the crystal structure of Cs_2_AgIn_0.9_Bi_0.1_Cl_6_ is shown in Figure 1f. Four main diffraction peaks were observed at 23.75°, 33.97°, 42.13°, and 48.04°, assigned to the (0 2 2), (0 0 4), (2 2 4), and (0 4 4) planes, respectively.^[^ ^11^ ^]^ The XRD peaks positioned between those of two pure phases (Cs_2_AgInCl_6_ and Cs_2_AgBiCl_6_) indicated that the substitution of In^3+^ with Bi^3+^ induced a gradual shift in the diffraction peaks toward a smaller angle (the fine structure analysis of the XRD pattern is shown in Figure S4 in the Supporting Information).^[^ ^13^ ^]^ In addition, the XRD results are consistent with theoretical calculations. To simplify the calculation model, Cs_2_AgIn_0.917_Bi_0.083_Cl_6_, a substitution compisiton close to the feeding ratio composition, was used (Figure S5, Supporting Information). We calculated the interplanar spacings (*d*) (listed in Table S1 in the Supporting Information) by XRD data, which yielded a lattice constant *α*  =  10.59Å (close to the simulation result of 10.51 Å). These results are also consistent with the TEM analysis.

**Figure 2:**
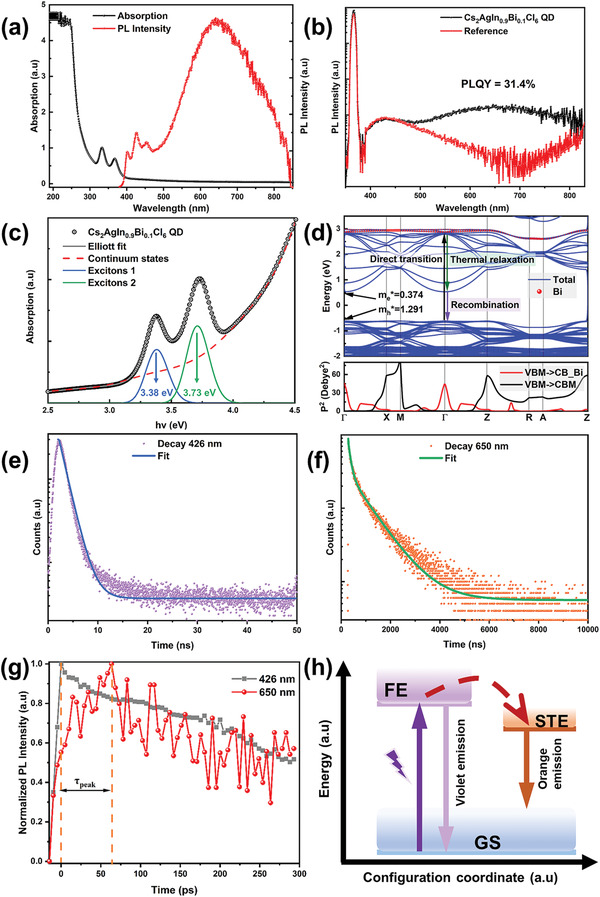
Basic optical absorption and emission characterization of the Cs_2_AgIn_0.9_Bi_0.1_Cl_6_ QDs. a) Absorption and PL spectra of the QDs, which shows obvious exciton behavior. b) Absolute PLQY of the QDs at an excitation wavelength of 375 nm. c) Absorption spectrum and Elliott's model of QDs for the energy range of 2.5–4.5 eV. The absorption spectrum (black circles) near the bandgap was fitted by the Elliott's model (gray line). The contributions of excitonic (blue and green lines) and continuum state (red dash line) transitions are also presented. The fitting is described in detail in Supporting Information. d) Calculated band structure (top) and transition matrix elements (bottom) of Cs_2_AgIn_0.917_Bi_0.083_Cl_6_. e,f) TRPL of two PL peaks of the QDs. The fluorescence emission (426 nm) with a short lifetime is from band‐to‐band transition, while the other emission at 650 nm is related to bound exciton states. g) Normalized PL decay curves within a very short time window measured by ultrafast‐picosecond spectrometry of two emission peaks. It clearly shows the concurrence of the decay at 426 nm and development at 650 nm exciton resonance. h) Configuration coordinate diagram for the exciton emission dynamic mechanism of QDs.

The absorption profile of Cs_2_AgIn_0.9_Bi_0.1_Cl_6_ QDs contains two exciton peaks at 333 and 367 nm, corresponding to two split valence bands with different effective mass of holes (**Figure** **2**a). The photoluminescence (PL) spectrum of the QDs shows an evident double‐color emission, with a relatively high absolute PLQY of 31.4% (Figure 2b). The Elliott model was used to describe the behavior of Coulombic interactions between electrons and holes, with a sharp exciton absorption (Figure 2c, gray line). The Elliott theory (see Supporting Information) elucidate the bimolecular recombination in perovskites and the broadening of absorption spectra.^[^ ^14^ ^]^ The absorption components of excitonic (blue and green lines) and continuum (red dashed line) transitions could be decoupled from the band‐edge spectral fitting. According to the extracted plots, the exciton resonances are centered at 3.38 and 3.73 eV, respectively. The bandgap (*E*_g_) of continuum states is 4.71 eV in the Tauc plot (Figure S6a, Supporting Information),^[^ ^15^ ^]^ which yields two exciton binding energies E_b1_ and E_b2_ of 1330 and 980 meV, respectively. These values are several times those of bulk materials (250 meV for Cs_2_AgInCl_6_). Likewise, the exciton binding energy of QDs is ≈4.5–7.4 times that of single crystal for the traditional inorganic semiconductor materials and perovskites (see Table S2 in the Supporting Information).^[^ ^5^, ^16^ ^]^ The ultrahigh exciton binding energy of Cs_2_AgIn_0.9_Bi_0.1_Cl_6_ QDs at room temperature led to their excellent luminescent properties. The Urbach energy of Cs_2_AgIn_0.9_Bi_0.1_Cl_6_ QDs was estimated to be ≈305 meV by the continuous state absorption data (Figure S6b, Supporting Information).^[^ ^17^ ^]^ Ultraviolet photoelectron spectroscopy measurement were performed to detect the energy band structure of Cs_2_AgIn_0.9_Bi_0.1_Cl_6_ QDs. As shown in Figure S6c in the Supporting Information, the highest occupied molecular orbital of Cs_2_AgIn_0.9_Bi_0.1_Cl_6_ QDs was calculated to be at ≈−7.69 eV. Using thses results combined with the *E*_g_ values obtained above, we derived the lowest unoccupied molecular orbital level of≈−2.98 eV.

To provide a better understanding of the absorption and recombination mechanism in Ag/In/Bi double perovskites, density functional theory (DFT) calculations based on the Perdew‐Burke‐Ernzerhof functional were performed (see Supporting Information for details). As shown in Figure 2d, Cs_2_AgIn_0.917_Bi_0.083_Cl_6_ exhibited a direct bandgap at the Γ point. The projected density of states (Figure S7a, Supporting Information) reveals that the valence band maximum (VBM) has Ag‐*d*, In‐*d*, and Cl‐*p* characteristics, while the conduction band minimum (CBM) is mainly attributed to Ag‐*s*, In‐*s*, and Cl‐*p* states. This leads to a large difference between the effective masses of electrons (0.374 m_0_) and holes (1.291 m_0_) (Table S3, Supporting Information). Notably, according to the transition matrix elements, the parity‐forbidden transition from VBM to CBM was observed. However, band symmetry analyses suggest that the parity‐forbidden transition is broken in the deep energy level of the conduction band owing to the contribution of the Bi atom. This explains the large energy difference between the absorption of the continuum states and the violet recombination emission. Figure S7b in the Supporting Information shows a schematic linking the distributions of the electrons and holes with the absorption.

Double‐color emissions hace been reported for perovskites. The narrow high‐energy peak likely originates from free excitonic emission, while the broad low‐energy peak is attributed to the formation of STEs, similar to that of CsPbI_3_.^[^ ^5^, ^6^, ^18^ ^]^ Splitting was observed for the high‐energy peaks of Cs_2_AgIn_0.9_Bi_0.1_Cl_6_ QDs. We believe that the three peaks contributing to the violet emission correspond to band‐to‐band transitions of continuous states and two types of excitons. STEs occur in various materials, such as alkaline earth fluorides, alkali halides, lead halides, lead perovskites, and lead‐free perovskites.^[^ ^5^, ^19^ ^]^ In contrast to free excitons, the STE emission spectrum is usually broad with a significant Stokes shift from the absorption of free excitons. Similar to small polarons, STEs can couple to a soft lattice and produce transient elastic structural distortions without inducing permanent defects. As reported in previous researches, upon light excitation, the strong Jahn‐Teller distortion and recombination of the excited‐state structure are caused by the change in the electronic configuration of the AgCl_6_ octahedron after the absorption of photons.^[^ ^5^, ^20^ ^]^ In particular, Ag‐Cl bonds are elongated in the axial direction but compressed in the equatorial plane. In the Cs_2_AgIn_0.9_Bi_0.1_Cl_6_ QDs, although the broad orange STE emission is attributed to excited‐state defects, it actually implies the temporary and recoverable change in the crystal structure.^[^ ^21^ ^]^ Time‐resolved photoluminescence (TRPL) results confirmed the different origins of the double‐color emission. The high‐energy emission exhibited a relatively quick decay (less than 2 ns), which can be fitted by a biexponential function with the majority of short‐lived component (Figure 2e). On the other hand, the lifetime of the broad orange emission was over 1 µs (Figure 2f). As shown in Figure 2g, a short time observation window was utilized to study the energy transfer between the two types of PL emissions. The fast decay of the high‐energy PL was well‐matched with the concomitant increase in the PL signal at low energy. This was considered an the evidence for the fast transfer from free excitons to STE states (Figure 2h). The self‐trapping depth (or difference in activation energy for carrier transfer between free excitons and STE states) of ≈50 ps was one of the main reasons for the large Stokes shift.^[^ ^22^ ^]^ Deeper STEs produce a lower‐energy PL and are characterized with a longer period to emit, which is compatible with the larger lattice distortions required for the lower‐energy PL.^[^ ^20^ ^]^

**Figure 3:**
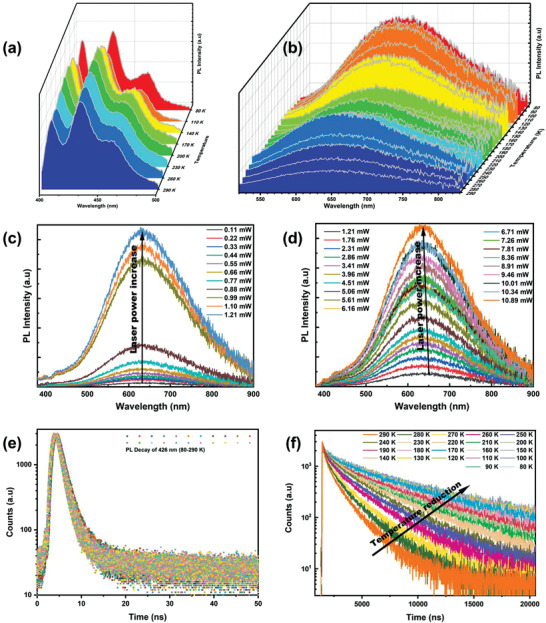
Temperature‐dependent PL properties of the Cs_2_AgIn_0.9_Bi_0.1_Cl_6_ QDs. a,b) Evolution of the PL intensity with the temperature reduction from 290 to 80 K for two emission peaks of the QDs. c,d) Power‐dependent PL plots. A picosecond diode laser in the continuous wave mode (375 nm, 1 MHz) was used as an excitation source. Limited by the detection capability of the detector, we properly increased the slit bandwidth when the excitation power was lower than 1 mW. A high‐pass filter (380 nm) was used to reduce the laser scattering. e,f) TRPL decay lifetime varying with the temperature reduction from 290 to 80 K for two emission peaks of the QDs. The different magnitudes of the two emission TRPLs show that their recombination mechanisms are disparate.

To determine the dynamics of the STEs, temperature‐dependent PL measurements were carried out. The STEs luminescence became dominant over the free exciton luminescence when the temperature decreased from 290 to 80 K (**Figure** **3**a,b). This trend has also been observed for pyrene and two‐dimensional perovskites, which is attributed to the reverse thermally driven transfer from STE to free exciton states and being tougher at lower temperatures.^[^ ^22^, ^23^ ^]^ The peak position of the STE luminescence exhibited a slight redshift, while that of the free exciton luminescence was unchanged when the temperature was reduced. Besides, the STEs peak profile became considerably narrower with the decrease in temperature, which indicates that vibrational coupling was responsible for the emission width.^[^ ^24^ ^]^ Further, the excitation intensity dependence of the STE emission was studied. The PL intensity increased with the laser power in the range of 0.11 to 11 mW at 300 K (Figure 3c,d), without sign of PL saturation. Notably, there is no change in the emission shape throughout the measurement, which suggests that the same emissive states are reached when the excitation intensity varies. The power dependence and insensitivity of the emission shape to the excitation intensity show that the excited‐state defects consisting of self‐trapped carriers are distinguishable from permanent material defects.^[^ ^24^, ^25^ ^]^ Upon cooling from room temperature, the PL decay lifetime of the free exciton emission fluctuated within a narrow range (Figure 3e; the parameters are listed in Table S4 in the Supporting Information). However, an apparent temperature dependence was observed for the PL decay lifetime of the STE emission (Figure 3f; the parameters are listed in Table S5 in the Supporting Information). The PL decay curve was considered to be the sum of fast and slow components, with a short lifetime of *τ*_1_ and a long lifetime of *τ*_2_. The obtained *τ*_1_ was approximately hundreds of nanoseconds. The proportion of *τ*_1_ decreased from 16.19% to 6.59% with the temperature decrease from 290 to 80 K. *τ*_2_ reached 6504.49 ns with a proportion of 93.41% when the temperature decreased to 80 K. Correspondingly, the whole PL decay lifetime was increased from 1148.81 to 6114.37 ns. The considerably prolonged *τ*_2_ indicates that the detrapping from STEs to free exciton states becomes more difficult at low temperatures.

**Figure 4:**
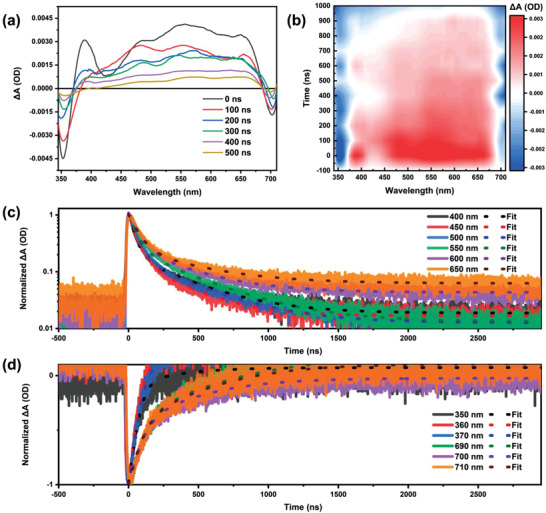
TA spectra of the colloidal Cs_2_AgIn_0.9_Bi_0.1_Cl_6_ QDs with a pump energy of 14 µJ cm^−2^ at 355 nm. a) TA plot at the indicated delay time from 0 to 500 ns, b) pseudocolor TA contour, c) PIA decay dynamics, and d) GSB kinetics of the Cs_2_AgIn_0.9_Bi_0.1_Cl_6_ QDs. The parameters of transient absorption kinetics are listed in the Supporting Information.

Transient absorption (TA) measurements were carried out to validate the origin of the long PL decay. Lead‐free perovskites with STE luminescence under near‐ultraviolet photoexcitation exhibit a broad photoinduced absorption (PIA) from an energy level below excitons expanding across the visible spectrum.^[^ ^5^, ^11^, ^20^, ^22^ ^]^ The TA plot and pseudocolor contour of the Cs_2_AgIn_0.9_Bi_0.1_Cl_6_ QDs (**Figure** **4**a,b) show a broad PIA band across the visible spectrum in the range of 370 to 690 nm, which confirms the presence of STEs. The TA signal lasted for hundreds of nanoseconds until carriers recombined. Only a positive signal was observed in the PIA decay dynamics (Figure 4c; the parameters are listed in Table S6 in the Supporting Information), which implies that only a direct transition occurs.^[^ ^11^ ^]^ Cs_2_AgIn_0.9_Bi_0.1_Cl_6_ QDs exhibit two ground‐state blench (GSB) peaks at ≈350 and ≈700 nm. The peak position is in agreement with the steady‐state absorption and TRPL results, which are related to the state filling of band‐edge excitons. These peaks were attributed to the transitions of a dual valence band structure.^[^ ^26^ ^]^ The GSB peaks exhibit an exciton‐like characteristic, which indicates that the initial photoexcitation electron‐hole pairs formed in the material are bound.^[^ ^27^ ^]^ The spectra in Figure 4a show the photoinduced blench as charge carriers recombine. By tracking the blench recovery of the corresponding transition, the recombination of photogenerated carriers in the Cs_2_AgIn_0.9_Bi_0.1_Cl_6_ QDs could be characterized. The normalized kinetic traces of GSB are presented in Figure 4d (the parameters are listed in Table S7 in the Supporting Information). It's apparent that the recombination rate is faster at short wavelengths. Figure S8 in the Supporting Information shows the TA plots of the Cs_2_AgIn_0.9_Bi_0.1_Cl_6_ QDs at varying pump intensities and excitation wavelengths. Notably, the specimen remained stable during the TA measurement. The rate of recombination increased with the pump fluence.

**Figure 5:**
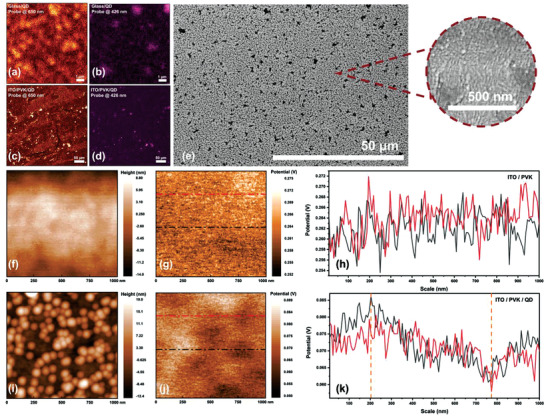
Morphology of the Cs_2_AgIn_0.9_Bi_0.1_Cl_6_ QD films. a,b) TRCI PL mapping of the QD films spin‐coated on a fused silica substrate with probe channels at 650 and 426 nm, respectively. c,d) TRCI PL mapping of the QD film deposited on an ITO/PVK substrate with probe channel at 650 and 426 nm, respectively. Fluorescence microscopy images were acquired with varying bandpass filters. e) SEM surface image of QD film on an ITO/PVK substrate (the inset shows a magnified view). f,i) Surface relative height difference map, g, j) relative CPD map, and h, k) CPD linescan spectra at two different positions of the ITO/PVK and ITO/PVK/QD films, respectively.

Further, the non‐toxic merit and bright orange emission of the Cs_2_AgIn_0.9_Bi_0.1_Cl_6_ QDs inspired us to fabricate WLEDs. It's known that traps can be introduced in the process of film formation from QD dispersions. Therefore, obtaining high‐quality thin films is an important step in the device fabrication. Here, PVK was selected as the hole‐transporting layer, and the optoelectronic properties of the deposited QD films were investigated. Serendipitously, violet radiation from PVK efficiently compensated for the deficit of violet emission from the Cs_2_AgIn_0.9_Bi_0.1_Cl_6_ QD thin film (Figure S9, Supporting Information). Time‐resolved confocal imaging (TRCI) results revealed that the QD film retained the same double color emission feature as in the PL results (**Figure** **5**a,b). When it was deposited onto the PVK‐coated substrate, the luminescent properties were unchanged (Figure 5c,d). Cs_2_AgIn_0.9_Bi_0.1_Cl_6_ QDs aggregated into islands on the substrates when the solvents were removed and exhibited a stronger luminescence. To elucidate the microscale morphology, scanning electron microscopy (SEM) images of the QD films were recorded, which showed a relatively smooth and compact QD film (Figure 5e). The distribution of island states was consistent with the TRCI results. According to the inset magnified view of the SEM image, the QD clusters were regularly arrayed on each QD island.

Subsequently, the electronic properties of the QD films were characterized using Kelvin probe force microscopy (KPFM). The resultant surface topography (Figure 5f) and contact potential difference (CPD) map (Figure 5g) of the PVK film exhibited a uniform trait. There is no difference between the extracted CPDs at each point from two different lines in Figure 5h. Compared to the PVK film, the QD film exhibited an increased roughness. The island aggregation is consistent with the results of the previous optical characterization (Figure 5i). In Figure 5j, the bright area exhibits a more positive CPD. In contrast, the dark area corresponds to a more negative CPD, which reveals that the CPD in the QD cluster gathering locatons is higher than that in the vacancy regions. This is further confirmed by the line‐scan spectra of different areas in the CPD profiles shown in Figure 5k. To further verify this result, a statistic analysis of the data of Figure 5g,j was carried out, as shown in Table S8 in the Supporting Information. The average CPD of the QD film was ≈71.2 ± 5.35 mV, 45 mV higher than that of the PVK film. Moreover, we marked the bright and dark areas in Figure 5j with numbers in green and blue, respectively (Figure S10, Supporting Information). The corresponding statistical results are summarized in Table S9 in the Supporting Information. The average CPD of the five marked green areas is ≈78.2 mV, While the CPD of the five marked blue areas is ≈63.64 mV. This indicates that charge carriers were locally confined within the high‐potential‐barrier island of Cs_2_AgIn_0.9_Bi_0.1_Cl_6_ QDs, which hinders the egress of carriers and is conducive to the generation of excitons. Therefore, the injected carriers cannot easily escape from QDs; instead, excitons can more easily recombine in QDs. In this regard, an enhanced luminescent efficiency is realized, which is one of the advantages of QD materials in LED applications.

**Figure 6:**
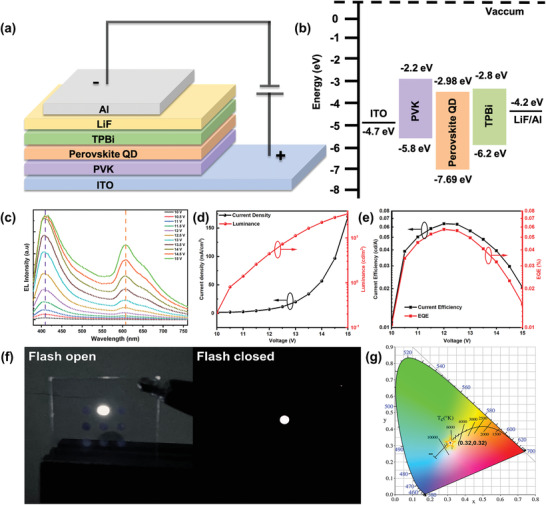
Cs_2_AgIn_0.9_Bi_0.1_Cl_6_ QDs based electroluminescence device. a) Illustration of the device structure (ITO glass/PVK/Cs_2_AgIn_0.9_Bi_0.1_Cl_6_ QDs/TPBi/LiF/Al). b) Energy band diagram of the Cs_2_AgIn_0.9_Bi_0.1_Cl_6_ QDs based WLED device. c) Electroluminescence spectra at varying bias voltages. d) Dependences of the current density and luminance on the driving voltage. e) Dependence of current efficiency and EQE on the driving voltage. f) Photographs showing the operating status (flash open and closed). g) CIE coordinates of the champion device.

A schematic of the QD‐WLED is shown in **Figure** **6**a. A QD‐WLED with a configuration of ITO/PVK/Cs_2_AgIn_0.9_Bi_0.1_Cl_6_ QDs/TPBi/LiF/Al was designed. The corresponding energy band diagram is presented in Figure 6b. The typical EL emission spectra of the QD‐WLED with the increase in forward‐bias voltage are shown in Figure 6c.The shape of the emission peaks was almost unaltered with the increase in the bias voltage. Current density–voltage (*J*–*V*) and luminance–voltage (*L*–*V*) curves of the QD‐WLED are shown in Figure 6d. The turn‐on voltage (at 1 cd m^−2^) and luminance value were measured to be 10 V and 34.7 cd m^−2^ (15 V), respectively. Figure 6e shows the current efficiency and EQE, with maximum values of 0.058 cd A^−1^ and 0.064%, respectively, at a bias voltage of 12 V. Photographs of the operating devices with bright and uniform white emissions are presented in Figure 6f. An operation video of the QD‐WLED under varying voltages is presented as Supplementary Video 2, which clearly shows the sustained, effectual, and luminous white EL with the increase in bias voltage without any packaging. The collected white emission corresponds to the CIE chromaticity coordinates of (0.32, 0.32), which belong to the typical warm white region as shown in Figure 6g. In particular, the correlated color temperature (CCT) and CRI of the QD‐WLED were determined to be 6432 K and 94.5, respectively, suitable for high‐definition display and lighting application. A slight change in CIE coordinates was observed in the operation stability measurement (Figure S11, Supporting Information), which might have resulted from the increasing temperature of the working device.^[^ ^7^ ^]^

To further enhance the device performance of the QD‐WLED, we used the cracks present on the QD films to passivate the unoccupied area and simultaneously reduce the leakage current. We selected a conductive organic molecule, triphenylphosphine oxide (TPPO), which has been used to passivate reduced‐dimension perovskite films.^[^ ^28^ ^]^ Trace TPPO with gradient concentration (TPPO:QD [wt%] = 1:20, 1:15, 1:10, 1:5) was doped into the QD precursors before spin‐coating (hereafter, the TPPO‐doped film is referred to as TPPO‐QD). The SEM images indicate that TPPO fills tiny interspaces between the QDs, which leads to a more compact and uniform active layer (Figure S12, Supporting Information). Owing to the reduced defects caused by TPPO passivation, the turn‐on voltage of the device was simultaneously reduced. The EL spectra of the TPPO‐QD WLEDs remained unaltered, which suggests that the addition of TPPO did not change the light‐emitting zone (Figure S13a, Supporting Information). The best TPPO‐QD device (TPPO:QD = 1:5) exhibited a promoted luminance of 158 cd m^−2^, with an optimal EQE of 0.08% at 9 V (Figure S13b,c, Supporting Information). The injected charge carriers were considered to recombine within the QDs, owing to the poor electrical conductivity of TPPO. Figure S13d in the Supporting Information shows the optimization mechanism of the TPPO doping. The optimization effect of the doping concentration is shown in Figure S13e,f in the Supporting Information. As the doping concentration of TPPO was increased, the device luminance and operating lifetime were increased simultaneously. However, when the weight ratio of TPPO exceeded 20%, the corresponding device could not operate. To assess the long‐term operating stability of the QD‐WLEDs, the luminance with varying TPPO concentration was monitored in ambient air with a constant driving current density of 15 mA cm^−2^. The best TPPO‐QD device (TPPO:QD = 1:5) exhibited a long half‐lifetime (*T*_50_) of 48.5 min, which is competitive with the reported lead‐based WLEDs.^[^ ^29^ ^]^ The parameters of the reported WLEDs based on the lead‐free double perovskites are summarized in Table S10 in the Supporting Information. The performances of the lead‐free perovskite WLEDs in this study were superior to those of their counterparts, particularly for the white‐emission CIE coordinates and CRI. Although further progress is required for practical applications, lead‐free perovskite QD‐WLEDs are promising for use in the next‐generation light‐emitting applications.

## 3 Conclusion

In summary, neatly structured lead‐free double perovskite Cs_2_AgIn_0.9_Bi_0.1_Cl_6_ QDs were synthesized using the hot‐injection method. Both spectroscopic characterization and DFT calculations demonstrated that the violet and orange emissions originate from free excitons and STEs, respectively. The capability of confining the charge carriers was further confirmed by microscopic measurements. The high PLQY of 31.4%, large exciton binding energy, and long STE decay lifetime make the Cs_2_AgIn_0.9_Bi_0.1_Cl_6_ QDs suitable as a light‐emitting layer in LEDs. We successfully fabricated electrically excited WLEDs based on the lead‐free double perovskite Cs_2_AgIn_0.9_Bi_0.1_Cl_6_ QDs for the first time. The QD‐WLED exhibited an excellent CIE color coordinates and CCT of (0.32, 0.32)/6432 K with a relatively high CRI of 94.5. A maximum luminance of 158 cd m^−2^ was achieved with a promoted EQE of 0.08% by TPPO passivation. This lead‐free QD‐WLED employed superior stability in ambient air with a long *T*_50_ of ≈48.53 min. The features of non‐toxic nature, facile solution processing, and outstanding stability enable the Cs_2_AgIn_0.9_Bi_0.1_Cl_6_ QDs next promising candidate for optoelectronic applications.

## Conflict of Interest

The authors declare no conflict of interest.

## Supporting information

Supporting Information

Supplemental Video 1

Supplemental Video 2

## Data Availability

The data that supports the findings of this study are available in the supplementary material of this article.
