# Supplementary material for: Lead‐free Double Perovskite Cs2AgIn0.9Bi0.1Cl6 Quantum Dots for White Light‐Emitting Diodes
Source: Adv Sci (Weinh). 2021 Nov 28;9(2):2102895. doi: 10.1002/advs.202102895 (PMC8805553; doi:10.1002/advs.202102895)
Supplement: Supplementary file 1 — Supporting Information [file ADVS-9-2102895-s002.pdf]

ADVANCED SCIENCE Open Access

Supporting Information

for *Adv. Sci.*, DOI: 10.1002/advs.202102895

Lead-free Double Perovskite $Cs_2AgIn_{0.9}Bi_{0.1}Cl_6$ Quantum Dots for White Light-Emitting Diodes

*Yuqing Zhang, Zehao Zhang, Wenjin Yu, Yong He, Zhijian Chen, Lixin Xiao, Jun-jie Shi, Xuan Guo, Shufeng Wang, Bo Qu**

Supporting Information

Lead-free Double Perovskite $Cs_2AgIn_{0.9}Bi_{0.1}Cl_6$ Quantum Dots for White Light-Emitting Diodes

*Yuqing Zhang [a], Zehao Zhang [a], Wenjin Yu [a], Yong He [a], Zhijian Chen [a], Lixin Xiao [a], Jun-jie Shi [a], Xuan Guo [a,b], Shufeng Wang [a], Bo Qu [a] **

[a] State Key Laboratory for Artificial Microstructures and Mesoscopic Physics, Department of Physics, Peking University, 100871, Beijing, People's Republic of China.

[b] Key Laboratory of Optoelectronic Science and Technology for Medicine of Ministry of Education, Fujian Provincial Key Laboratory for Photonics Technology, Fujian Normal University, Fuzhou 350007, PR China.

Email: bqu@pku.edu.cn

Experimental Section

Materials. Cesium acetate (Cs(OAc), 99.9%, metals basis), n-Hexane (97%), oleylamine (OLA), and LiF were purchased from Aladdin. Indium (III) acetate ($In(OAc)_3$, 99.99%, metals basis), silver acetate (Ag(OAc), 99.5%), and bismuth (III) acetate ($Bi(OAc)_3$, 99.99%, metals basis) were purchased from Alfa. Chlorotrimethylsilane (TMSCl), oleic acid (OA), methyl acetate (Me(OAc)), and octadecene was purchased from Macklin. Poly(9-vinylcarbazole) (PVK) were purchased from Lumtec. 1,3,5-Tris(1-phenyl-1H-benzimidazol-2-yl)benzene (TPBi) was purchased from Xi'an Polymer Light Technology Corp. (PLT). Triphenylphosphine oxide (TPPO, 98%) was purchased from Sigma-Aldrich. All these commercially available materials were used as received without any further purification.

Synthesis of $Cs_2AgIn_{0.9}Bi_{0.1}Cl_6$ quantum dots. Cs(OAc) (0.71 mmol), Ag(OAc) (0.5 mmol), $In(OAc)_3$ (0.45 mmol) and $Bi(OAc)_3$ (0.05 mmol) were dissolved in a combination of octadecene (10 mL), OA (2.5 mL), and OLA (660 μL), and this reaction mixture was heated to 110 °C under vacuum for 40 min. The mixed solution, which was initially colorless, gradually turned brown with the temperature increasing. The reaction mixture was then heated to 180 °C under a nitrogen atmosphere, and 0.5 mL of TMSCl was injected swiftly. After 30 s, the reaction mixture was cooled to room temperature in an ice-water bath. The reaction mixture was then centrifuged at 10000 rpm for 12 min. The darkly colored supernatant was decanted, leaving an orange-red precipitate. This material was taken up in n-hexane (10 mL)

and re-suspended with sonication. This mixture was then centrifuged at 8000 rpm for 15 min again, and the precipitate from this centrifugation was discarded. The obtained supernatant was transferred to another centrifuge tube and added extra Me(OAc) for purification. The final precipitate was collected *via* centrifugation and re-dispersed in n-hexane for further use. The obtained QD-dispersions were stored at 4 °C, and re-centrifuged at 8000 rpm for 15 min before each use.

Device fabrication. The ITO-coated glass substrates were washed sequentially in deionized water, acetone and ethanol for 30 min respectively, then treated with oxygen plasma for 10 min. A thin PVK (5.5 mg/mL in chlorobenzene) layer was deposited by spin-coating at 3000 rpm for 30 s, then annealed at 150 °C for 20 min. QDs (which were diluted to 1.5 mg/mL, and re-suspended with sonication for 30 min, then filtered by a 0.22 μm PES membrane before use) were deposited via spin-coating at 5000 rpm for 30 s in a N_2 glovebox, and anneal the QD film at 150 °C for 10 min. For the TPPO treated films, TPPO with gradient concentration (wt% TPPO:QD = 1:20, 1:15, 1:10, 1:5) were doped into QD precursors, and the deposition procedure was same as the pure QD films. TPBi (45 nm) and LiF (0.8 nm) were prepared by thermally evaporating method. Eventually, 100 nm aluminum anode was thermally evaporated on top of the devices through a shadow mask (the active area for all devices was 3.14 mm^2 as determined by the overlapping area of patterned ITO and Al electrode).

Characterization measurements. Surface morphology and cross-section images of QDs were characterized by transmission electron microscope (TEM, JEM-2100) and the energy dispersive spectra (EDS) mapping. The absorption spectra were recorded by a UV-Visible spectrophotometer (Shimadzu UV3600Plus). The X-ray diffraction (XRD) patterns were performed on a powder X-ray diffractometer (PANalytical Inc. X-Pert3 Powder) with monochromatic Cu Kα irradiation (λ= 1.5418 Å). The XRD pattern of theoretical simulation with the wavelength of 1.5418 Å was performed by VESTA package. Photoluminescence (PL), time-resolved photoluminescence (TRPL) spectra (excited at 375 nm) and the absolute photoluminescence quantum yield (PLQY) were measured by lifetime and steady-state spectrometer (FLS980 Edinburgh Instruments Ltd.). Time-resolved confocal imaging (TRCI) was measured by fluorescence lifetime imaging system (Q2 US. ISS. Rayleigh resolution R_{xy} = $0.61\lambda/NA \approx 200$ nm, NA = 1.2, excited at 375 nm. In order to isolate the needed signal, 641/75 nm and 460/80 nm bandpass filters were set in 650 nm and 426 nm probe channels, respectively.). The X-ray photoelectron spectra (XPS) and ultraviolet photoelectron

spectroscopy (UPS) patterns were performed by X-ray photoelectron spectrometer (AXIS Supra, Kratos Analytical Ltd.). The transient absorption spectra (TAS) were carried out on transient absorption spectrometer (LP980, Edinburgh Instruments Ltd). The ultrafast TRPL within a short time window were measured by LifeSpec red spectrometer (Edinburgh Instruments Ltd. Instrument response of 5 ps). Surface morphology of QD films were characterized by scanning electron microscope (SEM, Hitachi S-4800). The topography and surface potential of QD films were characterized by Kelvin probe force microscopy (KPFM) (multimode 8, Bruker Corp.). The current density and voltage characteristics of OLEDs were recorded by using a computer controlled Keithley 2611 Sourcemeter. The luminance characteristics of OLEDs were measured on an SRC-600 spectroradiometer (EVERFINE Corporation).

Elliott fitting. The absorption of a semiconductor near the band edge can be recounted by Elliott theory,[30] that is the energy-dependent absorption can be described by a combination of electron-hole continuum states and bound excitons:[31]

$$\alpha\left(E, E_g, E_b\right) = \frac{b_0}{E}\sum_{n=1}^{\infty}\frac{4\pi E_b^{3/2}}{n^3}\delta\left(E - \left[E_g = \frac{E_b}{n^2}\right]\right) + \frac{b_0}{E}\left[\frac{2\pi\sqrt{\frac{E_b}{E-E_g}}}{1-e^{-2\pi\sqrt{\frac{E_b}{E-E_b}}}}\right]c_0^{-1}JDoS(E) \quad \text{(S1)}$$

where $\alpha\left(E, E_g, E_b\right)$, E, E_g, and E_b represents absorption, energy, bandgap energy, and exciton binding energy respectively. The joint density of states is given by:

$$JDoS(E) = \begin{cases} c_0\sqrt{E - E_g}, & E > E_g \\ 0, & otherwise \end{cases} \quad \text{(S2)}$$

$c_0 = \frac{1}{2\pi}\left(\frac{2\mu}{\hbar^2}\right)^{\frac{3}{2}} \times 2$, is the joint density of states constant, where μ is the reduced effective mass of carriers.

Here, we broadly follow the treatment as Davies *et al.*[32] The absorption model can be illustrated as the sum of excitonic and continuum states convolved with a broadening function. The broadening function is a normalized function centered at the maximum and is the convolution of a normal distribution caused by electron-phonon coupling. In other words, the Elliott function can be described as:

$$f_{Elliott} = \alpha\left(E, E_g, E_b\right) \otimes N(0, \Gamma^2), \quad \otimes\ for\ convolution \quad \text{(S3)}$$

A least-squares minimization method was used to fit the absorption for the sample, with E_g fitted globally across data.

Urbach rule. Steep band edges were found in the absorption spectra of perovskites, which could be characterized by Urbach energy (E_0).[33] In the absorption plot, E_0 is defined as the slope of the exponential part. Here, the E_0 of double perovskite QDs in this work was calculated according to the Urbach rule:

$$\alpha_c = \alpha_0 e^{(h\nu - E^*)/E_0} \quad \text{(S4)}$$

where α_0 and E^*were material-dependent constants. The absorption coefficient α_c means continuum states absorption extracted from the whole experiment data.

Theoretical calculation. All the simulations were carried out using Vienna *Ab initio* Simulation Package (VASP) with the projector augmented-wave method within the framework of density functional theory (DFT).[34] To match the experimental measurement of lattice constants, the Armiento-Mattsson of generalized gradient approximation (GGA) was used to treat the exchange and correlation interactions for structural relaxation.[35] But Perdew-Burke-Ernzerhof (PBE) function was applied to calculate electronic properties.[36] Valence electrons for Cs, Ag, In, Bi and Cl were set to $5s^2 5p^6 6s^1$, $4d^{10} 5s^1$, $4d^{10} 5s^2 5p^1$, $5d^{10} 6s^2 6p^3$ and $3s^2 3p^5$, respectively. A cut-off energy of plane-wave basis set was set to 400 eV in all calculations. The Monkhorst-Pack scheme *k*-point grids of 2×4×4 and 4×8×8 were employed to geometry relaxation and electronic property calculations, respectively. The lattice and atomic positions were fully optimized with the convergence of 1.0×10^{-4} eV for total energy and 0.01 $eVÅ^{-1}$ for remainder force on each atom. Considering the limitation of computation resources, we construct a 3×2×2 supercell (containing 1 Bi and 11 In atoms) model to reproduce the $Cs_2AgIn_{0.9}Bi_{0.1}Cl_6$ quantum dots. Based on previous reports, the spin-orbit coupling (SOC) effect of In/Ag/Bi double perovskites is not sensitive to the bandgap type.[37] Hence, the SOC effect is not considered in our calculations to save the computing time.

TRPL fitting. The PL decay of $Cs_2AgIn_{0.9}Bi_{0.1}Cl_6$ QDs was fitted with a biexponential function using the equation given below:[38]

$$I(t) = A_1 e^{t/\tau_1} + A_2 e^{t/\tau_2} \quad \text{(S5)}$$

where τ_1 and τ_2 are two biexponential lifetimes with amplitudes of A_1 and A_2, respectively. The average lifetime "τ" was calculated using the parameter obtained from equation S4:

$$\tau = \frac{A_1 {\tau_1}^2 + A_2 {\tau_2}^2}{A_1 \tau_1 + A_2 \tau_2} \quad \text{(S6)}$$

For giving a better expression to the proportion of τ_1 and τ_2, the percentage of TRPL was calculated as follows:

$$\begin{cases} Rel_1 = \frac{\tau_2-\tau}{\tau_2-\tau_1} \times 100\% \\ Rel_2 = \frac{\tau_1-\tau}{\tau_1-\tau_2} \times 100\% \end{cases} \tag{S7}$$

This fitting method was also used in transient photoinduced absorption decay dynamics and transient absorption ground-state blench kinetics.

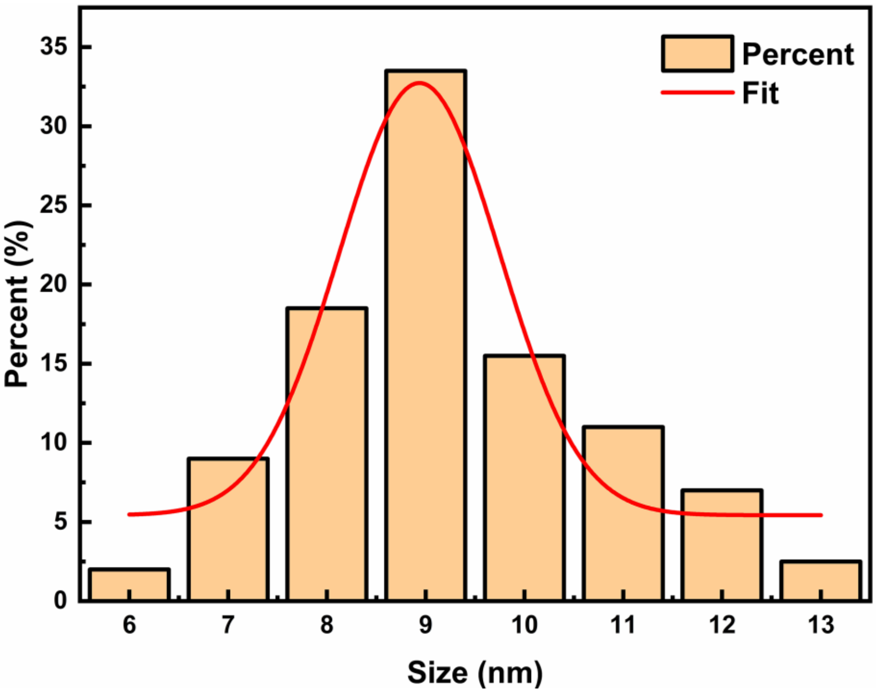

Figure S1 Size distribution estimated from low-magnification TEM image. This distribution was done using DigitalMicrograph software by taking 200 counts of selected QDs in **Figure 1**c.

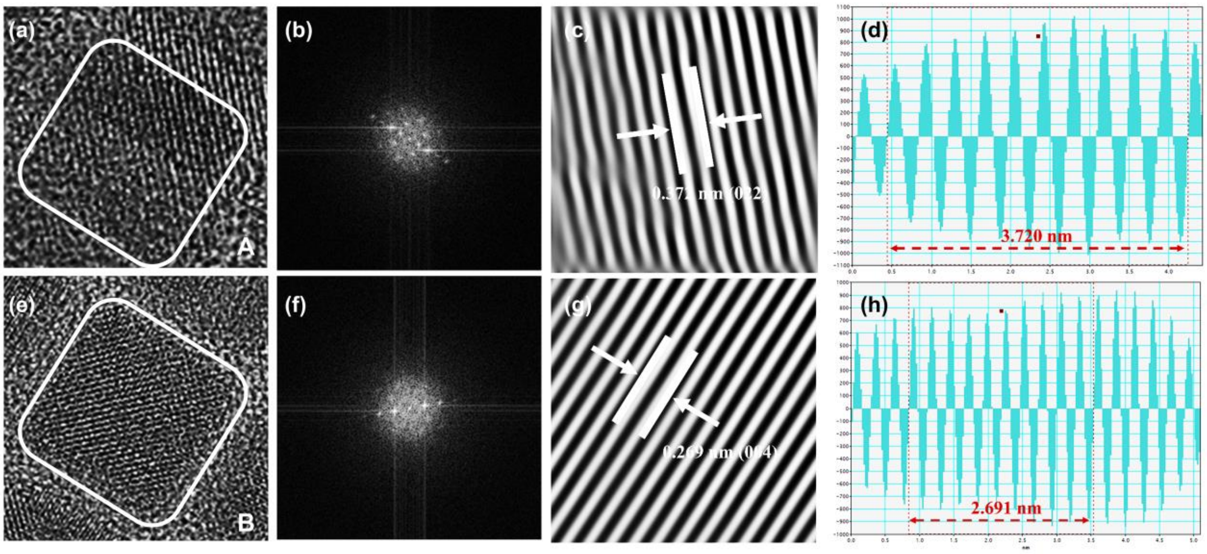

Figure S2 High-resolution TEM and corresponding FFT images of $Cs_2AgIn_{0.9}Bi_{0.1}Cl_6$ QDs depicting atomic lattice fringes, and the faceted cubic shape of the crystals. (a-d) are corresponding to crystal plane (0 2 2). (e-h) are corresponding to crystal plane (0 0 4).

Table S1. Interplanar spacing calculated according to XRD peak positions.

**2θ (deg)**	**Millerindices**			**d (nm)**
	**h**	**k**	**l**	
23.75	0	2	2	0.37
33.97	0	0	4	0.26
42.13	2	2	4	0.21
48.04	0	4	4	0.19

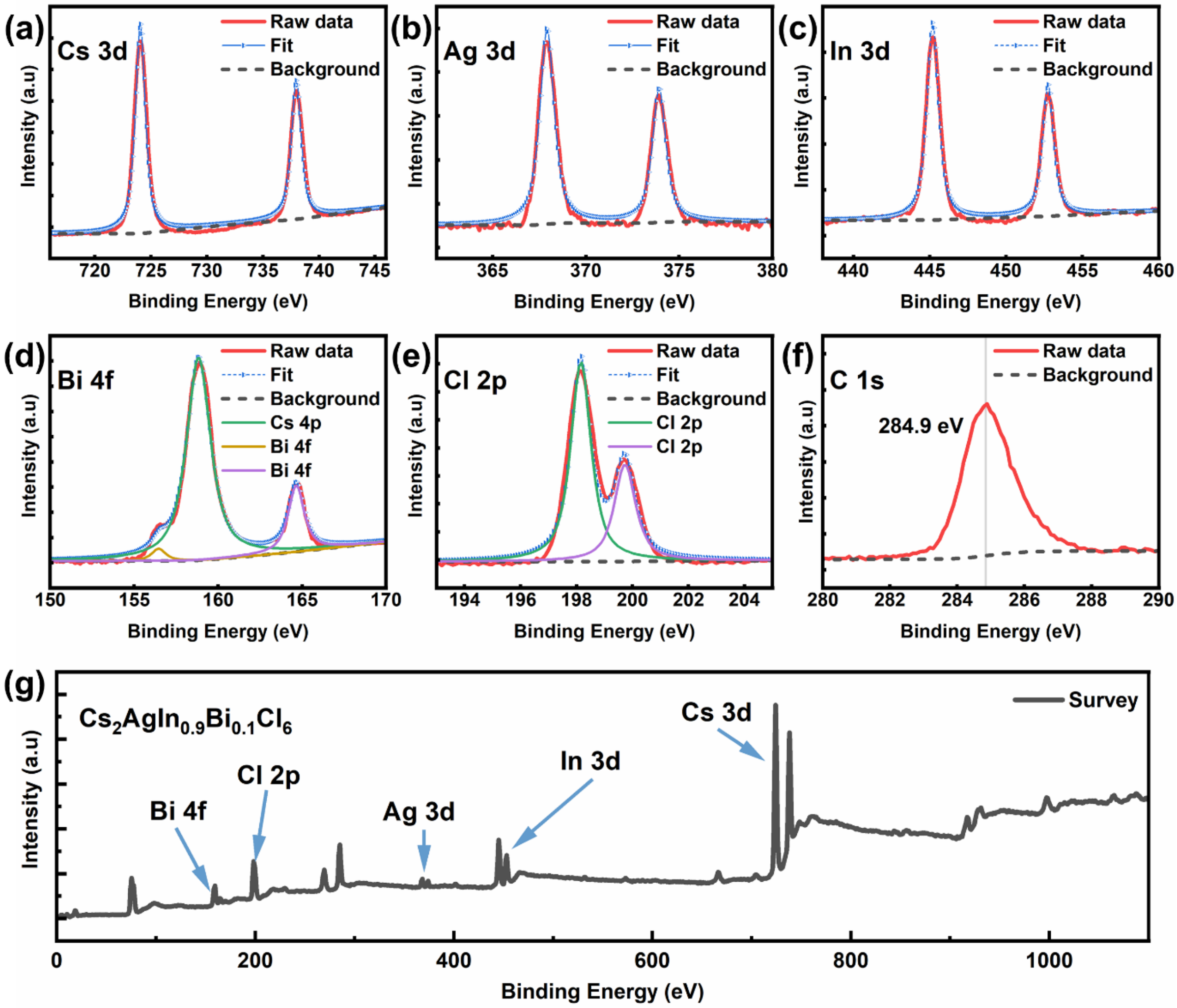

Figure S3 XPS images for different elements: (a) Cs 3d, (b) Ag 3d, (c) In 3d, (d) Bi 4f, (e) Cl 2p, (f) C 1s and (g) elemental analysis survey of $Cs_2AgIn_{0.9}Bi_{0.1}Cl_6$ QDs.

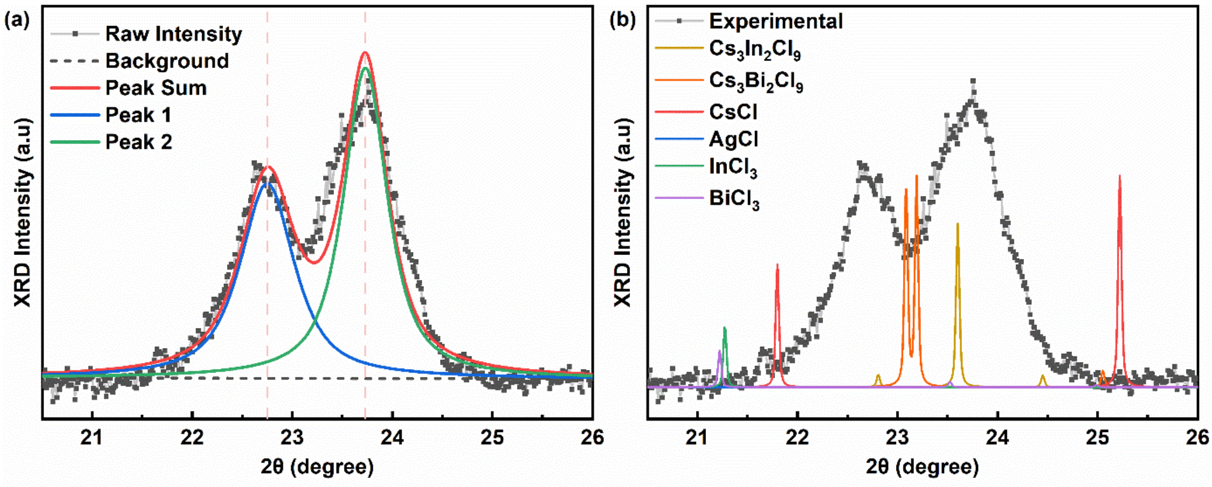

Figure S4 (a) Fine structure of the XRD pattern in **Figure 1**e. (b) XRD patterns of the possible decomposition products of $Cs_2AgIn_{0.9}Bi_{0.1}Cl_6$.

As shown in Figure S4a, a doublet was observed in the XRD patterns. The analysis for the fine structure of XRD patterns was added in the revision, as shown in Figure S4a. The peak analysis result indicates two kinds of crystal orientation in the specimen (diffraction peaks are located at 22.75° and 23.73°). According to the calculated results (23.96°), the peak at 23.73° can be assigned to the (0 2 2) plane. Three possibilities exist for the peak at a lower angle: (1) Owing to the uneven distribution of substituted elements, Bi-rich areas formed in local regions. (2) The peaks of decomposition products appeared in the test results. (3) Due to the weak periodic arrangement of the peripheral part of QDs, the lattice constant of these parts becomes larger, and the diffraction peak blue shifts accordingly.

The peak of pure $Cs_2AgBiCl_6$ locates at 23.35°, which is not consistent with experimental data. So the peak at 22.75° can not be considered evidence for the Bi-rich area's existence. For possible decomposition products in case of (2), the XRD patterns of $Cs_3In_2Cl_9$, $Cs_3Bi_2Cl_9$, CsCl, AgCl, InCl, $BiCl_3$ were calculated. As shown in Figure S4b, these peaks of the possible decomposition products were also inconsistent with the experimental data, which rules out the (2) situation. Therefore, we believe that the peak at the lower angle with an FWHM less than 0.5°, origins from the peripheral part of QDs, which has a larger lattice constant.

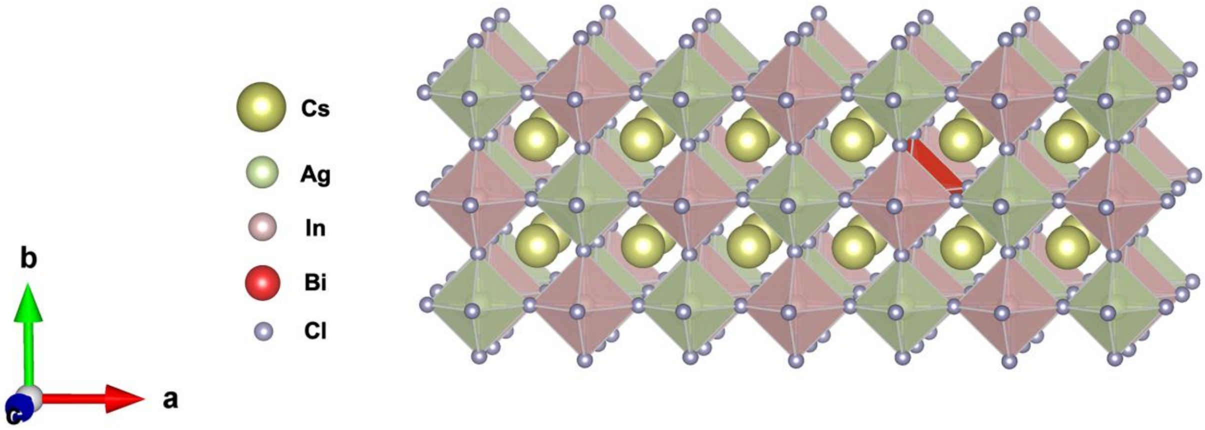

Figure S5 Crystal structure of $Cs_2AgIn_{0.917}Bi_{0.083}Cl_6$.

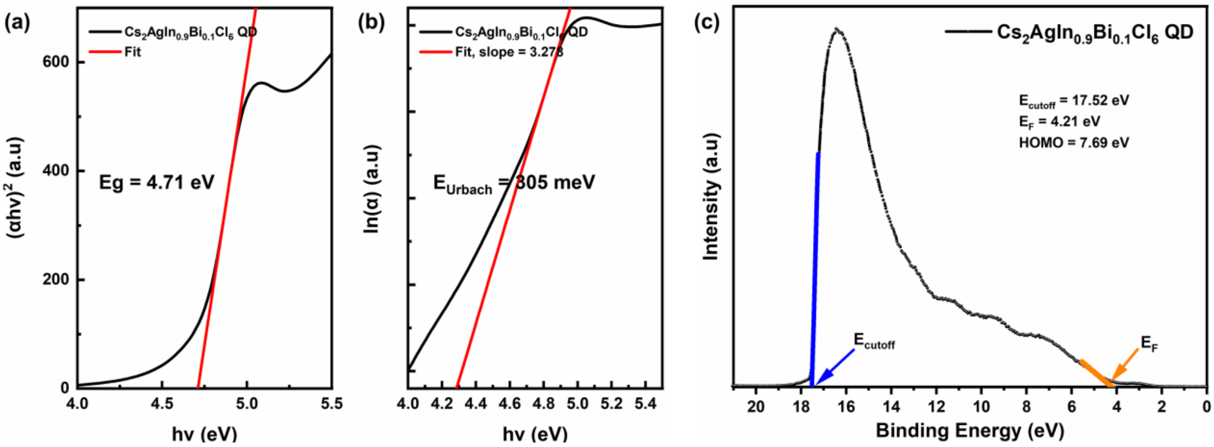

Figure S6 a) Tauc plot and (b) Urbach energy calculating results of energy band continuum states in $Cs_2AgIn_{0.9}Bi_{0.1}Cl_6$ QDs. (c) UPS results and the corresponding HOMO energy level of $Cs_2AgIn_{0.9}Bi_{0.1}Cl_6$ QDs.

Table S2. Summary of reported exciton binding energy in perovskites.

Materials	Dimension	E_b (meV)	Ref.
$MAPbI_3$	Bulk	11	[32]
		13	[39]
		37	[40]
$MAPbBr_3$	Bulk	40	[39]
		50	[14b]
		65	[41]
	Quantum dots	375	[41]
$(C_6H_{13}NH_3)_2PbI_4$	Layered bulk	400	[42]
	Quantum well	361	[43]
$(C_{10}H_{21}NH_3)_2PbI_4$	Layered bulk	370	[44]
$(C_4H_9NH_3)_2PbI_4$	Nanosheets	490 ± 30	[45]
$CsPbI_3$	Bulk	20	[46]
	Quantum dots	374	[47]
$CsPbBr_3$	Bulk	40	[46]
	Single crystal	38	[48]
	Quantum dots	198	[49]
$Cs4PbBr_6$	Nanocrystals	239.0 ± 34.8	[50]
$CsPbCl_3$	Single crystal	72	[48]
$CsSnI_3$	Bulk	12	[51]
$CsSnBr_3$	Bulk	19	[51]
$CsSnCl_3$	Bulk	37	[51]
$MA_3Bi_2Br_9$	Single crystal	57.1	[52]
$Cs_3Bi_2Br_9$	Single crystal	77.9	[52]
	Quantum dots	210.7	[52]
$Cs_3Cu_2I_5$	Single crystal	492	[53]
$CsCu_2I_3$	Bulk	202.5	[54]
	Single crystal	346.23 ± 14.12	[55]
$Cs_3Sb_2Br_9$	Quantum dots	530	[12b]
$Cs_2AgInCl_6$	Bulk	250	[5c]

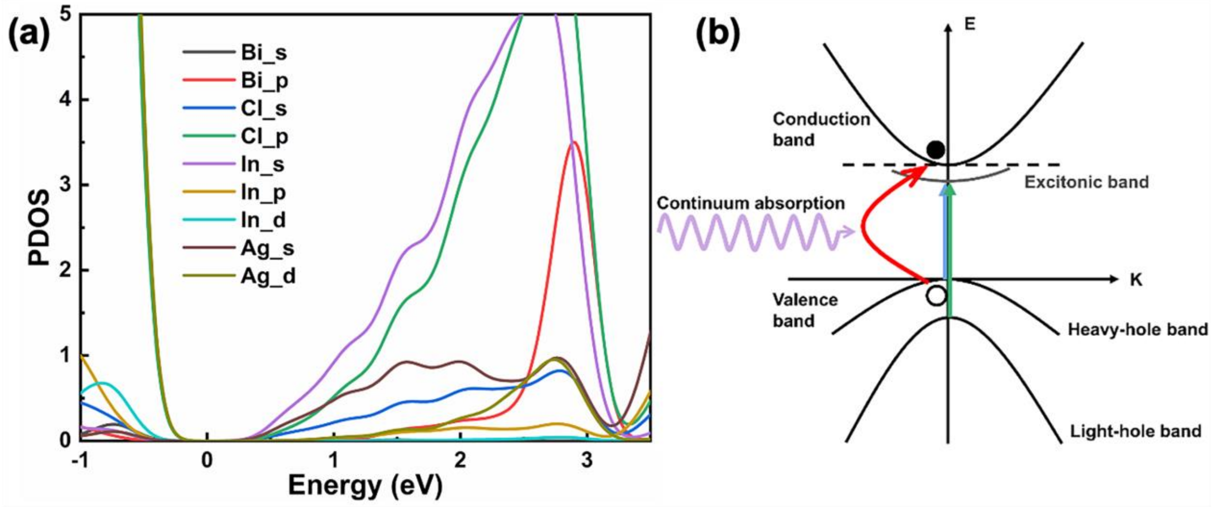

Figure S7 (a) Projected density of states (PDOS) of $Cs_2AgIn_{0.917}Bi_{0.083}Cl_6$, using PBE method. (b) Schematic diagram of the electron interband optical transition, which has a determining role in the bimolecular charge-carrier recombination process. The blue and green arrows from different valance bands are correspond to two exciton absorption peaks. The red arrow indicates continuum absorption.

Table S3. Effective mass of electrons and holes.

	**Electron (m_0)**	**Hole (m_0)**
G->X	0.449	1.864
G->Y	0.342	1.074
G->Z	0.342	1.074
Average	0.374	1.291

Table S4. Detailed parameters of temperature dependent TRPL at the emission of 426 nm.

Temperature (K)	τ_1 (ns)	Rel_1 (%)	τ_2 (ns)	Rel_2 (%)	τ (ns)
290	1.13	91.75	8.21	8.25	1.72
280	1.13	90.62	6.03	9.38	1.60
270	1.18	91.11	7.22	8.89	1.72
260	1.29	93.53	9.65	6.47	1.841
250	1.29	92.02	8.92	7.98	1.90
240	1.41	92.52	8.70	7.48	1.95
230	1.38	91.06	6.82	8.94	1.87
220	1.37	91.71	6.77	8.29	1.82
210	1.50	93.28	11.19	6.72	2.16
200	1.53	94.14	10.80	5.86	2.086
190	1.49	93.37	7.93	6.63	1.95
180	1.57	95.90	14.34	4.10	2.10
170	1.54	94.39	14.86	5.61	2.29
160	1.52	94.39	12.04	5.61	2.11
150	1.53	93.82	12.50	6.18	2.21
140	1.53	92.49	9.04	7.51	2.10
130	1.53	92.77	9.20	7.23	2.09
120	1.49	89.85	7.82	10.15	2.13
110	1.47	89.22	7.41	10.78	2.11
100	1.40	86.02	6.48	13.98	2.11
90	1.43	87.88	7.25	12.12	2.14
80	1.45	88.25	7.90	11.75	2.21

Table S5. Detailed parameters of temperature dependent TRPL at the emission of 650 nm.

Temperature (K)	τ_1 (ns)	Rel_1 (%)	τ_2 (ns)	Rel_2 (%)	τ (ns)
290	207.32	16.19	1330.66	83.81	1148.82
280	228.55	13.77	1606.79	86.23	1417.01
270	241.70	11.83	1842.36	88.17	1653.02
260	287.16	10.38	2293.51	89.62	2085.18
250	350.15	10.02	2689.67	89.98	2455.14
240	503.06	11.82	3248.73	88.18	2924.07
230	496.38	9.64	3619.05	90.36	3318.11
220	373.43	6.93	3868.67	93.07	3626.56
210	411.41	6.71	4346.24	93.29	4082.37
200	398.43	6.13	4657.31	93.87	4396.33
190	473.29	6.21	5079.65	93.79	4793.47
180	277.92	4.21	5289.25	95.79	5078.22
170	575.88	6.69	6018.33	93.31	5654.09
160	456.27	6.63	5684.04	93.37	5337.54
150	461.74	6.01	5904.98	93.99	5577.62
140	500.39	6.61	5999.58	93.39	5636.33
130	435.29	5.82	6005.70	94.18	5681.31
120	359.41	4.89	5930.40	95.11	5657.77
110	434.27	5.56	6073.83	94.44	5760.07
100	499.32	6.12	6229.87	93.88	5879.10
90	566.31	6.53	6337.55	93.47	5960.91
80	584.37	6.59	6504.49	93.41	6114.37

Table S6. Detailed parameters of transient photoinduced absorption decay dynamics.

Wavelength (nm)	τ_1 (ns)	Rel_1 (%)	τ_2 (ns)	Rel_2 (%)	τ (ns)
400	42.39	37.54	356.63	62.46	238.66
450	49.19	48.38	290.48	51.62	173.74
500	53.05	43.16	381.58	56.84	239.78
550	55.72	32.28	396.80	67.72	286.69
600	64.65	38.03	359.90	61.97	247.61
650	65.86	36.94	423.19	63.06	291.20

Table S7. Detailed parameters of transient absorption ground-state blench kinetics.

Wavelength (nm)	τ_1 (ns)	Rel_1 (%)	τ_2 (ns)	Rel_2 (%)	τ (ns)
350	39.09	72.44	299.24	27.56	110.78
360	31.86	66.30	145.03	33.70	70.01
370	35.22	63.34	145.35	36.66	75.60
690	66.79	34.99	479.95	65.01	335.37
700	63.04	38.51	371.18	61.49	252.50
710	61.58	33.36	414.12	66.64	296.51

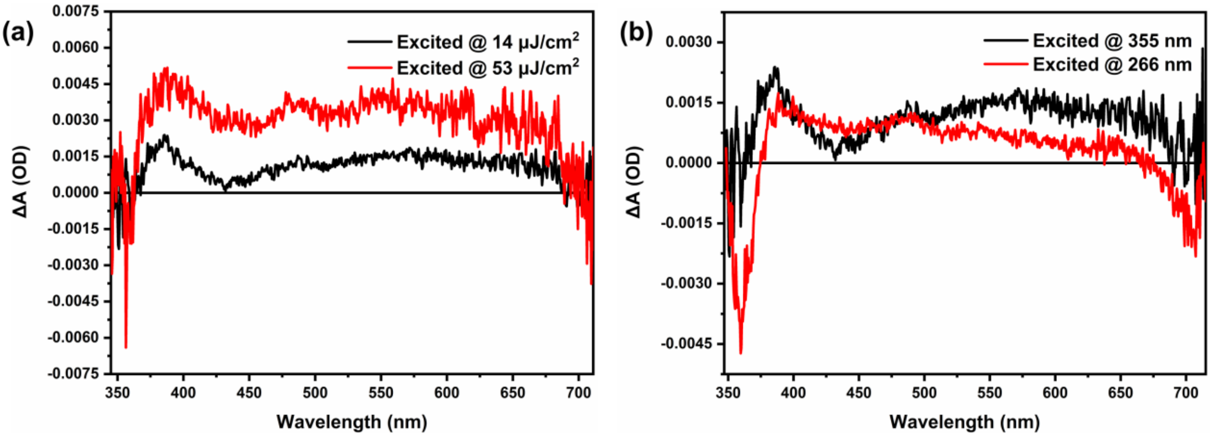

Figure S8 Transient absorption spectra (a) excited by 355 nm with different energy density, and (b) excited by different excitation wavelength with an energy density of 14 μJ/cm^2.

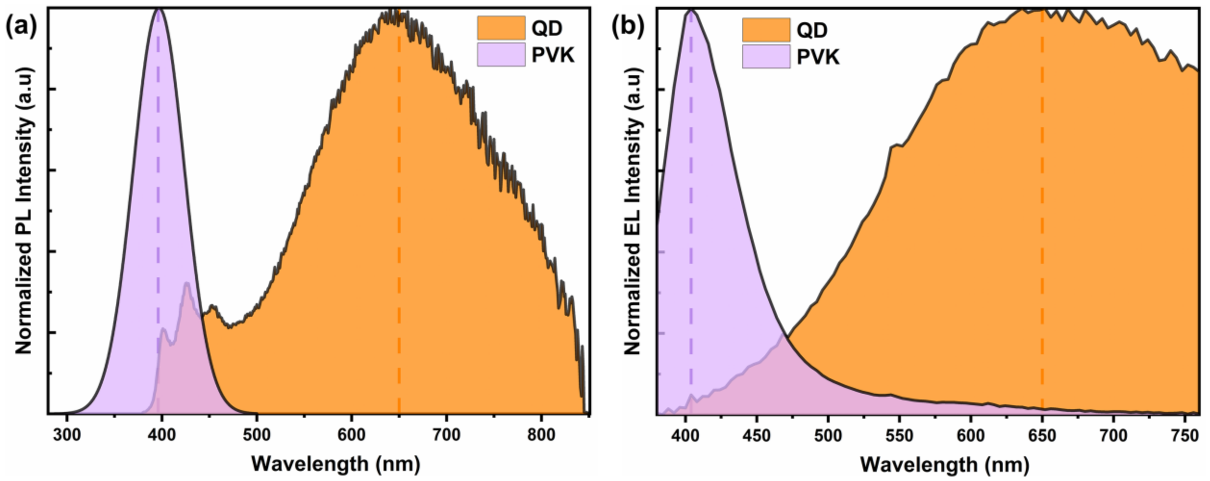

Figure S9 Normalized (a) PL and (b) EL (in visible region) spectra of $Cs_2AgIn_{0.9}Bi_{0.1}Cl_6$ QD and PVK thin films. In the PL measurement, the excitation wavelength was 375 nm, and all the specimen were coated on quartz glasses. The device structure used in EL measurement is ITO/PVK/TPBi/LiF/Al and ITO/QD/TPBi/LiF/Al, respectively.

Table S8 Statistical results of CPD map for PVK and PVK/QD films.

Sample	Average (mV)	Standard Deviation (mV)	Maximum (mV)	Minimum (mV)
PVK	26.2	3.52	27.8	24.9
PVK/QD	71.2	5.35	91.9	52.2

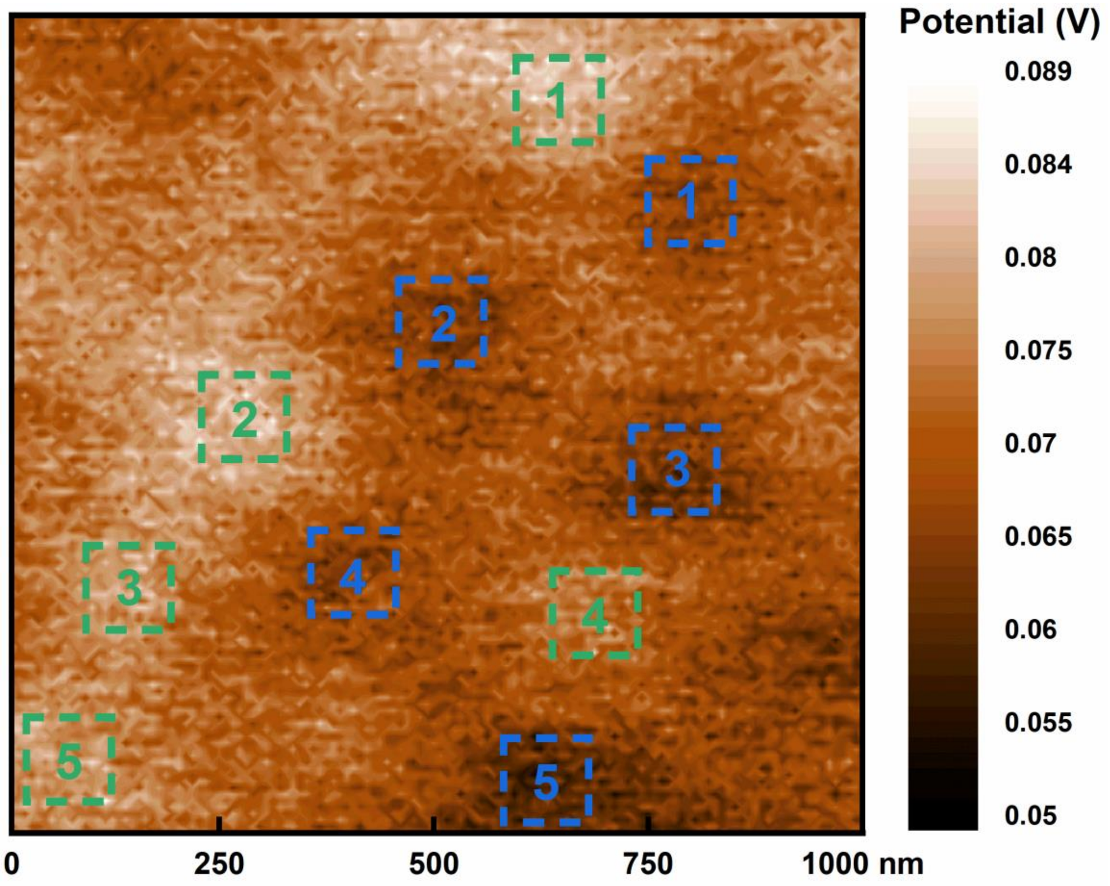

Figure S10 CPD map of PVK/QD film with marked areas. Five areas were selected from the bright and dark regions, respectively in Figure 5j (each selected area contains 100 data points). Furthermore, the Statistical results were summarised in **Table S**9.

Table S9 Statistical results of **Figure S**10.

Selected area	Average (mV)	Standard Deviation (mV)	Maximum (mV)	Minimum (mV)
Green 1	81.2	3.14	90.5	73.9
Green 2	80.4	3.29	88.9	70.5
Green 3	78.1	3.34	85.2	71.2
Green 4	73.8	3.21	84.6	71.3
Green 5	77.4	2.89	85.5	72.1
Blue 1	65.7	2.96	73.4	57.8
Blue 2	64.5	3.07	75.6	57.2
Blue 3	62.8	3.62	69.8	55.1
Blue 4	65.4	3.75	77.5	56.1
Blue 5	59.8	3.25	69.2	52.1

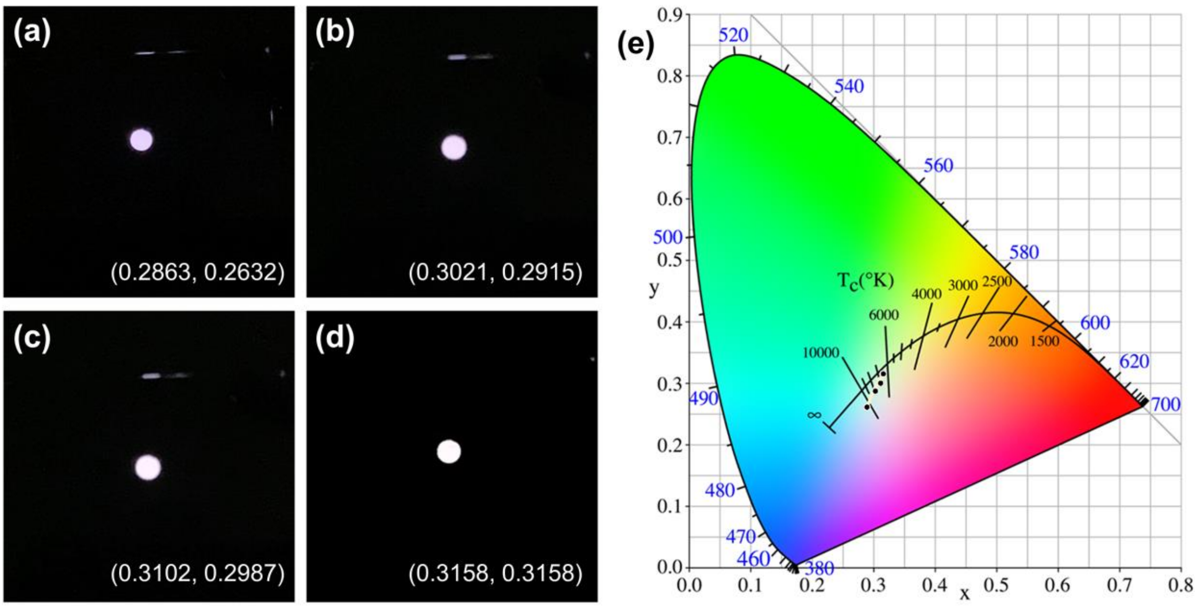

Figure S11 Evolution of CIE coordinates with voltage increasing.

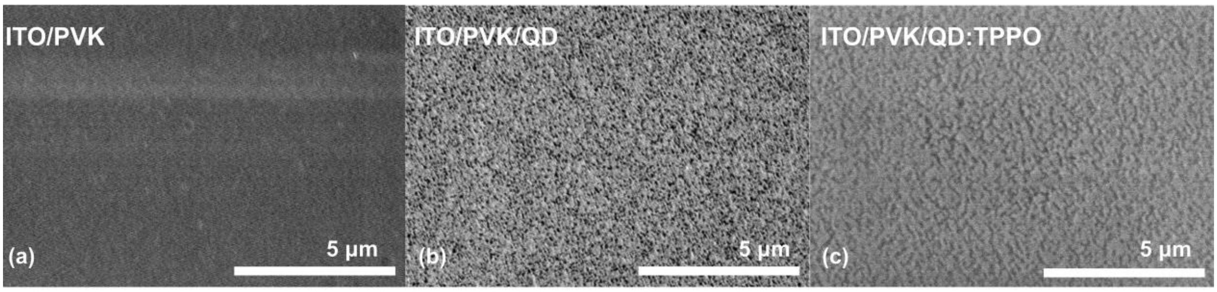

Figure S12 SEM surface morphology of (a) ITO/PVK thin film, (b) QD film on an ITO/PVK substrate, and (c) TPPO-QD film on an ITO/PVK substrate.

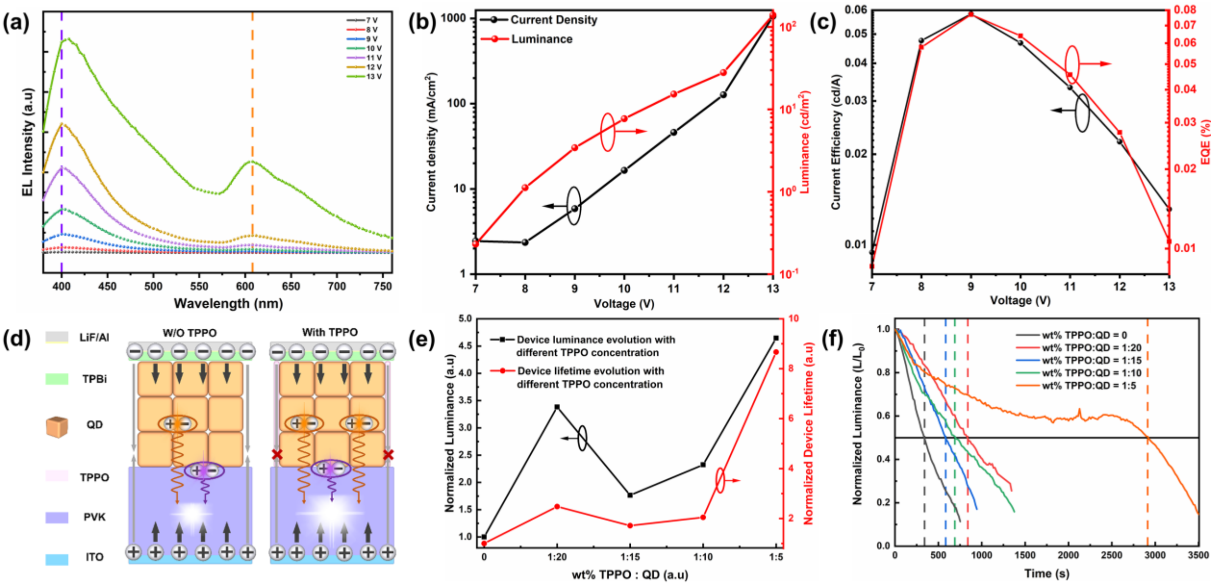

Figure S13 Device performances after TPPO optimization. (a) EL spectra, (b) Current density-voltage and luminance-voltage curves, and (c) current efficiency-voltage and external quantum efficiency-voltage curves of the device wt% TPPO: QD = 1:5. (d) Schematic representation of the TPPO optimization mechanism. (e) Device performances and (f) operation lifetime measured at 15 mA/cm^2 with varying TPPO concentrations.

Table S10. Summary of the device performance of WLED based on lead-free double perovskites.

	Emitter materials	PLQY (%)	CIE (x,y)	CCT (K)	CRI	L_{max}[a] (cd/m^2)	EQE (%)	Ref.
Electrically Excited	$Cs_2(Ag_{0.6}Na_{0.4})InCl_6$ Film	86 ± 5	(0.40, 0.45)	4054	-	~ 50	-	[5c]
	**$Cs_2AgIn_{0.9}Bi_{0.1}Cl_6$ QDs**	**31.37**	**(0.32, 0.32)**	**6432**	**94.5**	**158**	**0.08**	**This work**
Optically Excited	$Cs_2AgIn_{0.883}Bi_{0.167}Cl_6$ powders	62.1	(0.42, 0.39)	3260	87	-	-	[56]
	$Cs_2Ag_{0.6}Na_{0.4}InCl_6$ powders	86 ± 5	(0.40, 0.45)	4054	82	-	-	[5c]
	$Cs_2AgIn_{0.7}Bi_{0.3}Cl_6$ powders	87.2	(0.38, 0.44)	4347	87.8			[57]
	$Cs_2AgIn_{1-x}Bi_xCl_6$ NCs	10	(0.36, 0.35)	4443	91	-	-	[10]
	$(OCTAm)_2SnBr_4$ powders	95 ± 5	(0.33, 0.31)	6530	89	-	-	[58]

[a] L_{max} : maximum luminance
